# Better understanding complex pathomechanisms in central nervous system disorders as a prerequisite for improved diagnostic and therapeutic approaches

**DOI:** 10.1002/nep3.70017

**Published:** 2025-09-29

**Authors:** Piotr Walczak, Shen Li, Xunming Ji, Johannes Boltze

**Affiliations:** ^1^ Center for Advanced Imaging Research, Department of Diagnostic Radiology and Nuclear Medicine University of Maryland Baltimore Maryland USA; ^2^ Department of Neurology and Psychiatry, Beijing Shijitan Hospital Capital Medical University Beijing China; ^3^ Beijing Institute of Brain Disorders Capital Medical University Beijing China; ^4^ Chinese Academy of Medical Sciences and Peking Union Medical College China; ^5^ School of Life Sciences University of Warwick Coventry UK

**Keywords:** Acquired brain injury, astrocytes, endothelial cells, exosomes, ischemic stroke, major depressive disorder, mitochondrial transfer, myalgic encephalomyelitis/chronic fatigue syndrome, stem cells

1

Central nervous system (CNS) disorders are usually characterized by a complex pathophysiology. The last issue of *Neuroprotection* featured reviews and research articles looking at peripheral factors such as the gut microbiome^1^, ^2^ or a history of pre‐eclampsia^3^ and their impact on CNS conditions. Articles presented in the current issue of *Neuroprotection* will shift the focus back to the CNS but will continue to provide insights from recent research that help to better understand the pathophysiological complexity of CNS conditions.

Neuroinflammation is known to be a hallmark and major contributor to many CNS diseases. It comprises both peripheral and central immune cells and can be modulated by frequent comorbidities such as hypertension.^4^ Moreover, neuroinflammatory processes can involve cell populations not primarily characterized as immune cells. Psychiatric conditions, including major depressive disorders (MDD), are increasingly recognized to be linked to neuroinflammation^5^ but also to systemic comorbidities, stress and aging. A better understanding of neuroinflammatory processes in the context of psychiatric conditions may help to identify novel therapeutic targets for better and potentially causal treatment strategies. When focusing on neuroinflammation, microglia have been extensively investigated, while less is known about other cerebral cell populations involved in the regulation of neuroinflammatory processes. The systematic review by Singhal et al. summarizes the current knowledge about the role of astrocytes in neuroinflammation in the context of MDD. Recent research indeed revealed a central role of astrocytes in the pathophysiology of depression, comprising numerous aspects including promotion of neuroinflammation and reduction of synaptic plasticity. Peripheral inflammatory conditions and chronic stress can promote astrocyte transformation into a reactive phenotype with pro‐inflammatory functions. Moreover, reactive astrocytes and microglia can join to create a self‐sustaining pro‐inflammatory feedback loop, resulting in a vicious circle driving neuroinflammation. The review provides information about region‐specific astrocytic roles in MDD, elucidates phenotype‐depending responses and provides detailed mechanistic insights. The presented data underlines the importance of astrocyte‐driven neuroinflammation as a promising therapeutic target in MDD,^6^ particularly when working towards precision medicine approaches.

Acquired brain damage, for instance following traumatic brain injury or stroke, is a leading reason for permanent disability both in child‐ and adulthood. While causal therapeutic options after brain injury are still very limited, brain plasticity can help in regaining at least some lost neurological functions, contributing to better outcome. On the other hand, maladaptive plasticity exists and contributes to poor functional outcome in the long run. The review by Rajan provides a detailed overview on post‐injury adaptive plasticity in the mammalian brain. Hippocampal neurogenesis, generally known to be important for functional recuperation after acquired brain injury,^7^ is a beneficial mechanism that contributes to maintaining and regaining cognitive function while synaptogenesis can help to strengthen damaged neuronal circuits. The underlying mechanisms are explained in detail by this review with specific emphasis on long‐term potentiation and depression. Moreover, the role of axonal sprouting as a major element of post‐injury brain repair is discussed, including the main molecular mechanisms promoting or inhibiting axonal sprouting. The review further puts a spotlight on neuroinflammation. Neuroinflammation is a double‐edged sword in the context of adaptive plasticity following brain injury. While it can contribute to the early reparative process, prolonged neuroinflammation can hinder functional recuperation. The review explains mechanisms and mediators behind the dual role of neuroinflammation, which is of particular interest for therapeutic research. Finally, recent developments in the fields of neuromodulation techniques and pharmacotherapy to optimize adaptive plasticity after brain injury are discussed in detail.

Ischemic stroke induces widespread and rapid damage to all cerebral cell populations in areas of disrupted blood supply. There are multiple mechanisms of cellular damage in ischemic stroke with mitochondrial damage being increasingly recognized as a major contributor. Mitochondrial damage is an important pathomechanism as it will not only impair the production of energy‐containing substrates after potential recanalization, but also because it is a mediator of apoptosis. Meng and coworkers provide a detailed review about mitochondrial transfer that can counteract mitochondrial damage in ischemic stroke. The review provides insights into mitochondrial transfer processes explaining transfer mechanisms such as tunneling nanotubes and extracellular vesicles. Interestingly, mitochondrial transfer might be conducted by stem and progenitor cell populations, including mesenchymal stem cells (MSCs). MSC are known to improve stroke outcome in experimental models without replacing lost brain tissue, and direct cell‐cell interaction has been shown to be important for these therapeutic effects.^8^ The review continues with an explanation of potential beneficial effects of mitochondrial transfer in stroke focusing on metabolic support, anti‐inflammation, and antioxidation. It also provides an outlook on potential therapeutic application although the knowledge of how exactly mitochondrial transfer can be used to improve stroke outcome is still limited. Given to multiple beneficial effects on processes also related to ischemic reperfusion injury, it could represent a way to improve the outcome of clinically applied recanalization treatments, and research in this area has been recommended as a future priority.^9^

Despite inconclusive results of recent clinical studies, stem cell‐based therapies are still of interest as an experimental treatment for ischemic stroke. The enthusiasm for stem cells as therapeutic agents is partially driven by the very broad spectrum of potential beneficial effects many stem cell populations can exert, ranging from immunomodulation and antioxidative functions^10^ over plasticity enhancement to potential neurorestauration. Du and co‐workers contribute a comprehensive review paper summarizing the current knowledge of stem‐cell mediated therapeutic mechanisms after stroke. Importantly, the review also covers mechanisms mediated by exosomes, which are increasingly discussed as therapeutic alternatives to classical stem cell approaches. It also focuses on bioengineering strategies to enhance the therapeutic effects and provides detailed insights into the results of recent clinical trials. Importantly, the work also covers current clinical trials focusing on exosomes in ischemic stroke, although results from these studies have not been published yet. Thus, the review by Du et al. is a meaningful and compact resource for researchers using stem cells and exosomes as experimental stroke treatments.

The COVID‐19 pandemic has not only significantly reshaped the way we work and live but also reshaped research on neurodegenerative disorders. Many long‐lasting COVID‐19 infection sequelae comprise changes in neurological function and mental wellbeing, which are not well understood so far. A condition termed Long COVID, or post‐COVID syndrome, is characterized by symptoms including shortness of breath, muscle and joint pain, problems to memorize or focus appropriately, and strong fatigue. The symptoms are similar to those observed in myalgic encephalomyelitis/chronic fatigue syndrome (ME/CFS), a frequent yet poorly understood condition which is believed to involve metabolic and immunologic‐inflammatory pathomechanisms in multiple organ symptoms. The work by Herman presents a concept based on the similarities between long COVID and ME/CFS. Recent research suggests that both conditions include oxidative stress as a central pathomechanism, and the concept therefore suggests that Long COVID and ME/CFS may be used as models to better understand other neurodegenerative disorders based on excessive oxidative stress. The paper is clear about the fact that research in this area is just emerging and still in its infancy with many core hypotheses and pathomechanistic elements awaiting confirmation and verification. Nevertheless, it also provides intriguing perspectives for further research avenues as well as concrete examples for potential next steps.

Blood‐brain barrier (BBB) integrity is an important aspect of brain health. The BBB dysfunction is a frequent cause or consequence of brain injury. However, BBB permeability may also increase during aging.^11^ The reasons for this are incompletely understood. Guo et al. provide interesting in vitro data investigating the crosstalk between young (low passage) and senescent (high passage) astrocytes and endothelial cells. Young astrocytes induced the expression of barrier genes in coculture, actively contributing to decreased endothelial permeability. Senescent astrocytes did not have such effects. Moreover, it was found that senescent astrocytes express higher levels of angiotensinogen, an angiotensin precursor. Angiotensin is known to compromise BBB integrity. Reducing angiotensinogen expression in senescent astrocytes by siRNA restored the endothelial barrier‐promoting effects of senescent astrocytes. In turn, no influence of endothelial cell age on astrocytes was found with respect to angiotensinogen expression. Thus, the paper by Guo et al. offers novel insights into BBB biology in the context of aging.

This issue of *Neuroprotection* features a series of review and research highlight articles on a wide range of topics, as well as an original report on BBB permeability during aging. Together, the presented work contributes to better and more detailed understanding of the complex pathomechanisms of several CNS conditions while at the same time providing thought‐provoking concepts, ideas and working hypotheses for downstream research. The spectrum of CNS disorders and conditions discussed is particularly wide in the current issue. This reflects the broad scope of *Neuroprotection*. We anticipate that the work presented will be useful to colleagues in many fields of experimental neuroscience, and hope that it will also inspire future research projects.

## AUTHOR CONTRIBUTIONS

All authors listed have conceptualized, written and edited the publication, and approved its final version for submission.

## CONFLICT OF INTEREST STATEMENT

X.J., P.W., and J.B. are Co‐Editors in Chief of Neuroprotection. S.L. serves as an executive editor for the journal. All authors were blinded from reviewing or making decisions on the manuscript. The article was subject to the journal's standard procedures, with peer review handled independently of the Co‐Editors in Chief and their research groups. P.W. is a founder and holds equity in IntraArt, LLC and Ti‐com, LLC.
